# Modified Trochanteric Flip Osteotomy in Varus Intertrochanteric Osteotomy for Treatment of Legg–Calvé–Perthes Disease

**DOI:** 10.3390/children12010051

**Published:** 2025-01-01

**Authors:** Andrea Laufer, Carina Antfang, Georg Gosheger, Adrien Frommer, Gregor Toporowski, Henning Tretow, Robert Roedl, Bjoern Vogt

**Affiliations:** 1 Pediatric Orthopedics, Deformity Reconstruction and Foot Surgery, University Hospital Muenster, 48149 Muenster, Germany; 2 General Orthopedics and Tumor Orthopedics, University Hospital Muenster, 48149 Muenster, Germany

**Keywords:** Legg–Calvé–Perthes disease, trochanteric flip osteotomy, varus intertrochanteric osteotomy, release cut, blade plate

## Abstract

**Background/Objectives**: Legg–Calvé–Perthes disease (LCPD) presents challenges in treatment due to its varied course and unclear etiology. This study aimed to evaluate the efficacy of combining proximal femoral varus osteotomy (PFVO) with a modified trochanteric flip osteotomy to address biomechanical consequences and improve hip abductor muscle strength. **Methods**: We present a modified approach combining PFVO with a trochanteric flip osteotomy. In this technique, the greater trochanter in compound with its muscular insertions is separated from the femur and attached distally using a varization blade plate. Nine patients (ten hips, mean age 8 years) with LCPD were treated using this technique. Clinical examination findings and radiographic evaluations were retrospectively analyzed. The median follow-up was 33 months. **Results**: At the last follow-up, two patients exhibited Trendelenburg gait, but hip abduction was improved in all patients. Radiographically, consolidation at the osteotomy site was observed in all cases with no delayed union or non-union. The median CE angle improved by 7°, while the median CCD decreased by 18°. The median MPFA decreased by 13°, resulting in a median of 82°. **Conclusions**: Combining PFVO with a modified trochanteric flip osteotomy addresses biomechanical issues associated with PFVO, potentially improving hip containment and abductor muscle strength. This approach may offer advantages over traditional osteotomy techniques in treating LCPD, and it appears to produce a superior functional outcome in particular in regard to limping when compared to conventional PFVO. Despite satisfactory radiological outcomes in most cases, further research is needed to assess long-term effectiveness and address challenges such as femoral head enlargement and persistent gait abnormalities.

## 1. Introduction

Legg–Calvé–Perthes disease (LCPD) is an idiopathic osteonecrosis of the developing hip caused by the interruption of blood supply to the femoral head [1]. The avascular osteo- or chondronecrosis of the capital femoral epiphysis can cause severe deformity of the proximal femur and may ultimately lead to secondary osteoarthritis [2]. The etiology of the disease still remains mostly unclear [1,3]. It appears that mechanical as well as biological and environmental factors contribute to the development and may also be responsible for the heterogeneous epidemiologic and clinical features of this condition [1,2,4]. The varying and often unpredictable course of the disorder causes controversies upon the most adequate treatment regimen. Severe courses frequently lead to progressive deformation of the proximal femur resulting in early-onset osteoarthritis irrespective of the chosen treatment regimen [2,5]. On the other hand, intermediate courses of LCPD, presenting head-at-risk signs, do appear to benefit from surgery [6]. The main aim of treatment is preserving hip containment to retain the femoral head within the acetabulum during the period of regeneration of the capital femoral epiphysis as well as preservation of the blood supply of the femoral head to maintain the spherical shape and prevent progressive deformation [6,7]. The restoration of containment may either be achieved through pelvic or proximal femoral varus osteotomy (PFVO). Triple pelvic osteotomy is widely performed and has been reported to produce satisfactory results in regard to hip containment [8]. However, it should be noted that pelvic osteotomies bear a greater perioperative risk compared to PFVO [9]. Furthermore, LCPD is primarily a condition affecting the proximal femur, and it should preferably be addressed at the origin of the deformity. However, traditional PFVO has been associated with significant biomechanical impairments, and it has been criticized that containment treatment by PFVO disregards potential long-term sequelae [10]. Apart from producing leg length discrepancies through femoral shortening, the greater trochanter is inevitably proximalized, resulting in an overriding greater trochanter and, consecutively, insufficiency of the hip abductor muscles, which frequently leads to Trendelenburg gait [11,12]. Moreover, an overriding greater trochanter induces mechanical stress to the hip joint and may thus contribute to increased pressure to the femoral head, which may result in further chondral damage [13]. However, the aforementioned biomechanical consequences of an overriding greater trochanter may be alleviated by greater trochanteric transfer to restore abductor muscle strength, resulting in improved gait [14,15,16]. We thus combined PFVO with a distalization of the greater trochanter through a modified trochanteric flip osteotomy [17]. The greater trochanter in conjunction with the hip abductor muscles and the vastus lateralis is reattached in a distalized, adequate anatomical position in relation to the femoral head. The specific modification of this technique consists in threading the greater trochanter with the blade plate before fixating the proximal femur in a varus position. We hypothesize that this novel technique may decrease the risk of developing Trendelenburg gait by maintaining hip abductor muscle strength.

## 2. Materials and Methods

### 2.1. Patients and Indications

Since 2018, nine patients (ten hips) (seven males, two females) were treated with a modified trochanteric flip osteotomy combined with varus intertrochanteric osteotomy at a mean age at surgery of 8 years (range, 5 to 11). A chart review was performed to retrospectively evaluate clinical examination findings and clinical course. Median follow-up was 33 months (7–70) with one patient lost to follow-up after the first postoperative control.

**Figure 1:**
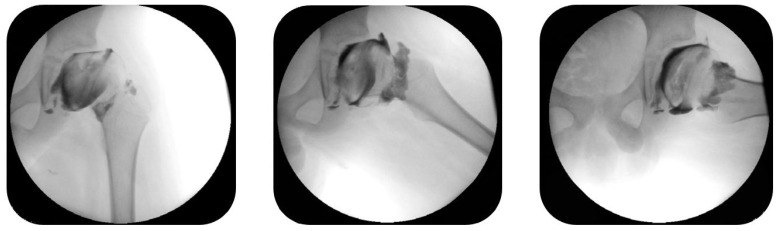
Dynamic arthrography of the hip was performed in all patients preoperatively to rule out a hinge abduction phenomenon. In the arthrograms above (patient 5; see Table 1), no hinge abduction could be detected.

Surgical treatment was considered in all children with LCPD who either presented a deterioration of the condition clinically (increasing pain, restricted range of motion, limping) or if a radiological tendency toward a more severe course of deformity with head-at-risk signs and Herring group B, B/C or C were observed. All children had a dynamic arthrography before the surgical intervention to detect a potential hinge abduction phenomenon (Figure 1). If hinge abduction was detected, surgical treatment was not conducted.

All X-rays were taken in a supine position adapting the angulation to take the pelvic tilt into account. Calibration markers were used as a standard.

### 2.2. Surgical Technique and Follow-Up Protocol

All procedures were performed by two senior surgeons (R.R., B.V.), who are also authors of this study.

**Figure 2:**
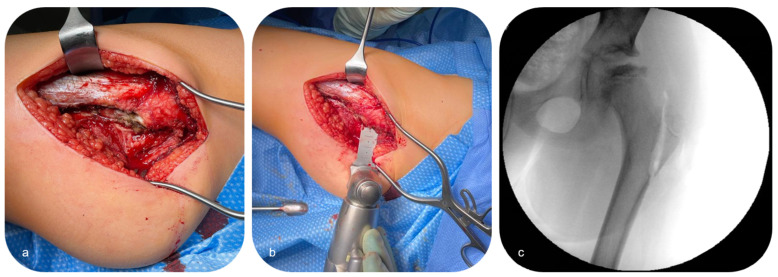
Lateral approach to the proximal femur with a fascia lata split (**a**) and vertical osteotomy at the base of the greater trochanter (**b**,**c**) (Patient 3; see Table 1).

The patient was initially placed in lateral decubitus position. A standard lateral approach to the proximal femur was performed, and the fascia lata was split to visualize the posterior aspect of the greater trochanter (Figure 2a). The latter was than separated from the proximal femur in compound with the attachment of the gluteus medius and vastus lateralis muscles through a vertical osteotomy with an oscillating saw (Figure 2b,c) before it was distalized under abduction of the hip. Excessive medial penetration of the proximal femur with the saw and osteotome, respectively, was avoided to prevent the risk of injury to the medial femoral circumflex artery.

**Figure 3:**
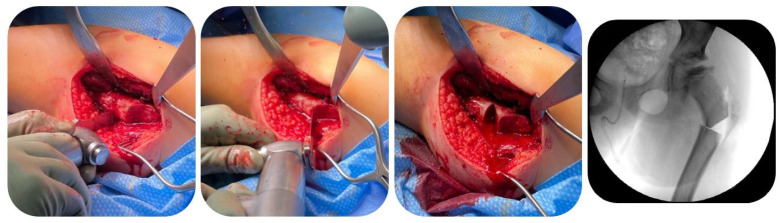
Recess cut at the proximal femur to achieve sufficient medialization of the distal fragment (Patient 3; see Table 1).

Furthermore, to protect the soft tissues including the medial circumflex artery, subperiosteal retractors were placed circumferentially around the osteotomy site. Subsequently, a recess cut was carried out for medialization of the distal fragment, as the offset of the blade plate was completely filled by the distalized greater trochanter (Figure 3).

**Figure 4:**
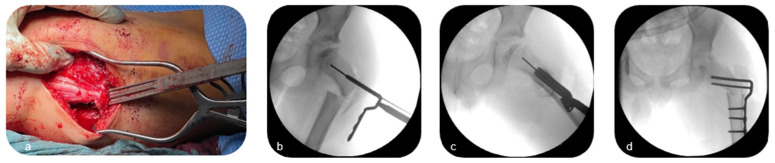
After preparation of the implant site with a cannulated chisel (**a**), a cannulated 90°- or 100°-blade plate is inserted over a guide wire (**b**,**c**), threading the distalized greater trochanter and fixating it to the femoral neck (**d**) (Patient 3; see Table 1).

Then, the patient was moved to supine position. A guide wire was then introduced into the femoral neck approaching from the distalized greater trochanter under consideration of the preoperatively determined correction angle. After radiographical verification of the correct wire position, the seating chisel and chisel guide were inserted over the guide wire, and the seating chisel was impacted to the appropriate depth (Figure 4a). After removal of the chisel guide, the osteotomy was performed at the proximal femur to achieve the predetermined angular correction, after which the blade plate (90° or 100°; OrthoPediatrics, IN, USA; Figure 4b,c) was inserted over the guide wire. Thus, the blade threaded through the distalized greater trochanter and fixated it to the femoral neck while fixating the plate to the bone in a varus position by locking screws (Figure 4d).

Postoperatively, weight bearing was completely restricted for six weeks. Physiotherapy was initiated during the hospital stay and was recommended at least once per week in the outpatient setting. Follow-up appointments were scheduled six weeks postoperatively and in three-month intervals from then.

### 2.3. Preoperative and Postoperative Clinical and Radiographic Evaluation

**Figure 5:**
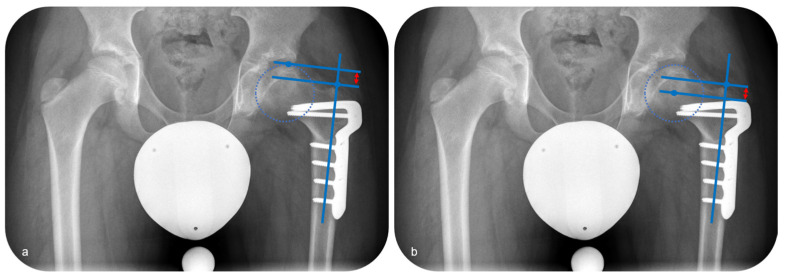
Measurement of the articulo-trochanteric distance ((**a**); red arrow) and the center head-trochanteric distance ((**b**); red arrow).

Anteroposterior and axial radiographs of the hip were analyzed preoperatively and at the last follow-up. The radiological evaluation was performed with regard to the stage of disease according to Waldenstroem, joint orientation and axis alignment with reference to established parameters [18]. The lateral pillar was assessed according to the Herring classification. For the children who reached the final stage of LCPD, the evaluation of the sphericity of femoral head was performed, taking the Stulberg classification into consideration. Concerning joint orientation, the medial proximal femoral angle (MPFA), the center-edge (CE) angle and the caput-collum-diaphyseal (CCD) angle were evaluated. The position of the tip of the greater trochanter in regard to the center of the femoral head was determined measuring the articulo-trochanteric distance (ATD) (Figure 5a) as well as the center head-trochanteric distance (CHTD) (Figure 5b).

## 3. Results

### Clinical and Radiographic Results

At the time of surgery, radiographs showed initial stage in one hip, condensation stage in four hips, and fragmentation stages in five hips. Herring group B was determined in two cases, group B/C in four cases, and group C in four cases. Catterall class II was observed in one case, class III in one case, and class IV in eight cases (Table 1). Limping was present in four patients. Hip abduction was clinically limited to 10 degrees or less in two patients. Yet, the performed arthrography in anesthesia of these two patients showed a noticeably better abduction ability.

At the time of the last follow-up, two of eight patients presented Trendelenburg gait. The median hip abduction was 38 degrees (range, 20 to 50). The median limb length discrepancy (LLD) was 0 mm (range, 0 to 20) in the total group and 15 mm (range, 15 to 20) in the three patients with measured LLD. Radiographically, all patients presented sufficient consolidation at the osteotomy site. There were no cases of delayed union or non-union nor implant failure. Two hips presented Stulberg group II, and one hip presented Stulberg group V. Six hips were in the reparative phase according to Waldenstroem.

The median MPFA was 100° (range, 83 to 103) preoperatively and 82° (range, 72 to 105) postoperatively with a median decrease of 13° (range, 3 to 29). The median CE angle was 15° (range, 6 to 23) preoperatively and 22° (range, 12 to 32) postoperatively with a median improvement of 7° (range, 0 to 20). The CE angle improved in eight of ten hips, while it did not change in two hips. The median CCD was 142° (range, 130 to 147) preoperatively, and 118° (range, 104 to 130) postoperatively, with a median decrease of the CCD of 18° (range, 11 to 41). The ATD increased in five and decreased in five hips, respectively. The median ATD was 17 mm (range, 15 to 24) preoperatively and 16 mm (range, 6 to 29) postoperatively. The median CHTD was 6 mm (range, 3 to 10) preoperatively and 8 mm (range, 0 to 12) postoperatively with a median increase of 2 mm (range, −4 to 6).

At the time of the last follow-up, we had found no osteonecrosis of the femoral head in any of the treated hips. Implants had been removed in six of ten hips. The median time between initial surgery and implant retrieval was 17 months (range, 10 to 45).

## 4. Discussion

Since its introduction in 1965 [18], PFVO has become an established surgical treatment approach for LCPD [19]. By producing increased varus angulation of the proximal femur, the femoral head is centered within the acetabulum [20]. To further improve containment, the proximal femur may additionally be altered in the sagittal and transverse plane through modifications of the osteotomy [21]. The aim is to shift the fragile capital femoral epiphysis out of the main load zone [21]. The load-relieving effect may ultimately improve remodeling of the biologically plastic femoral head during the healing or revascularization phase [20,21]. Moreover, relocation of the femoral head through PFVO does not influence the acetabular center of rotation, as is the case in pelvic osteotomies. Thus, an increase of the intraarticular pressure and further impairment of the blood supply to the femoral head can be avoided [22].

PFVO has shown to achieve radiographic results—in particular regarding the Stulberg classification—superior to those of patients undergoing non-operative treatment [19]. Nonetheless, the correct indication and timing of PFVO are crucial to achieve a satisfactory outcome. Containment should be re-established before the onset of re-ossification of the femoral head [23]. If conducted in a timely manner, PFVO may help to shorten the fragmentation phase, and femoral head deformation may be less severe due to the reduced lateral subluxation of the femoral head [5,23]. Ideally, the spherical shape of the femoral head can be preserved. In particular, patients older than six years of age should be considered for surgical treatment, as the ability of the femoral head to spontaneously remodel seems to remarkably decrease beyond this age [24]. This patient group also appears to benefit more from PFVO than non-operative treatment [5,20].

Even though PFVO has been reported to be beneficial to the healing process if indication, timing and technique are executed appropriately, it is also associated with certain pitfalls and disadvantages. For instance, preservation of the medial femoral circumflex artery is of utmost significance to maintain sufficient blood supply to the proximal femur and prevent further damage [10]. Furthermore, the overcorrection of varus angulation may shift part of the femoral neck into the main load zone, resulting in incorrect load distribution, which may impair healing of the capital femoral epiphysis in a spherical shape [21]. It is generally recommended to not produce a varus angulation of less than 100° [25,26]. However, even the moderate varization of the proximal femur will inevitably produce an elevation of the tip of the greater trochanter relative to the center of rotation of the femoral head. An overriding greater trochanter, in turn, results in shortening of the origin to insertion length and an increased lever arm of the hip abductor muscles [27]. The decreased resting length and abductor lever arm ratio may lead to a functional insufficiency of the hip abductors [28]. Owed to this, Trendelenburg gait as well as fatigue pain on walking are frequently observed after PFVO and may persist permanently [29]. It is described that up to 46% of patients develop limping after conventional PFVO [11]. Moreover, an overriding greater trochanter may also cause painful pelvitrochanteric impingement, further limiting hip joint mobility [30].

Combining PFVO with a modified trochanteric flip osteotomy to distalize the greater trochanter addresses these issues by re-establishing the original resting length and lever arm, thus maintaining hip abductor strength. The necessity of subsequent surgeries may hence be avoided by preserving or improving hip function, pain, and gait. The principle and rationale of the PFVO with trochanter distalization for the treatment of LCPD was first presented by Birke et al. at the European Paediatric Orthopaedic Society meeting 2016 [31]. Birke combined a trochanter flip approach and development of the retinacular soft tissue flap with a novel release cut technique to achieve varization with the necessary medialization of the femoral shaft. He utilized a 130-degree cannulated blade plate allowing sufficient bone stock for refixation of the distalized greater trochanter, which was fixated at the lateral aspect of the plate. We modified this technique by employing a 90- or 100-degree rather than a 130-degree blade plate, which allows threading the greater trochanter with the blade and fixating it to the femoral neck. However, since the threaded greater trochanter impedes sufficient medialization through the blade offset, a recess cut is still required to avoid lateral translation of the distal fragment. Even though radiologically, adequate distalization of the greater trochanter was observed after PFVO with a modified trochanter flip osteotomy, we nevertheless observed persistent Trendelenburg gait in two patients. This may be due to the fact that we performed only moderate lateralization of the greater trochanter. However, apart from height of the greater trochanter, an adequate distance between the center of the femoral head and the tip of the greater trochanter also plays an important role regarding the effectiveness of abductor muscle strength, since the pelvitrochanteric muscles require less force to maintain the pelvis level during the single stance phase [32]. In LCPD, enlargement and flattening of the femoral head is frequently observed, thus reducing the CHTD. This may lead to a weakening of hip abductor muscle strength, even if the greater trochanter has been sufficiently distalized [28]. In patients preoperatively presenting an enlarged femoral head and Trendelenburg gait, more aggressive lateralization of the greater trochanter should thus be considered to improve hip abductor strength. Alternatively, a femoral neck lengthening osteotomy has been declared effective to achieve this goal while concomitantly decreasing leg length discrepancy [28]. However, it should be noted that femoral neck lengthening osteotomy inevitably increases the intraarticular pressure and may thus aggravate deformation of the femoral head in LCPD. Apophyseodesis of the greater trochanter has been proposed as another alternative approach to PFVO to prevent an overriding greater trochanter and, consecutively, Trendelenburg gait [10]. This procedure may also avoid iatrogenic varus deformity [33]. However, it has been constituted that this measure only renders effective if performed before eight years of age and if Trendelenburg gait has not yet established [34,35]. Apart from age at surgery, it appears that the size of the femoral head at healing significantly influences the effectiveness of apophyseodesis of the greater trochanter [36], thus further limiting its applicability. We thus believe that in patients in whom this technique is not applicable, PFVO combined with a modified trochanter flip osteotomy is an efficient procedure to restore the physiological position of the greater trochanter, keeping its biomechanical function as a hip stabilizer while improving hip containment. Even though we observed an unsatisfactory radiological outcome with Stulberg Class V hip in one patient of the studied cohort at the time of last follow-up, it should be noted that this patient showed Herring class C preoperatively, which reportedly is associated with a poorer treatment outcome irrespective of age at and choice of treatment [20]. Moreover, the pathogenesis of LCPD is immensely complex, and it may thus not always be sufficient to solely modify biomechanical factors [5].

This study has a few limitations, for no real control group exists to compare the development of Trendelenburg gait in children who underwent surgery with a trochanteric flip osteotomy and those without. Furthermore, an observer bias could exist, as gait was assessed during consultation by varying doctors. An assessment using standardized gait analysis could improve the objectivity.

## 5. Conclusions

Despite satisfactory radiological outcomes in most cases, further research is needed to assess long-term effectiveness and address challenges such as femoral head enlargement and persistent gait abnormalities, in particular regarding Trendelenburg gait. This modified technique represents a promising approach to preserve hip function and prevent complications associated with traditional PFVO.

## Figures and Tables

**Table 1:** Patient characteristics with pre- and postoperative clinical and radiological outcome parameters. m = male, f = female, n/a = not available, Preop. = preoperative, Postop. = postoperative, F = fragmentation, C = condensation, I = initial, R = reparation, CE = center-edge, CCD = caput-collum-diaphyseal, ATD = articulo-trochanteric distance (ATD), CHTD = center head-trochanteric distance.

Patient	Age at Time of Surgery (Years)	Sex	Preop. Limping	Preop. Herring Stage	Preop. Catterall Stage	Preop. Waldenstroem Stage	Postop. Waldenstroem Stage	Postop. Stulberg Grade	Preop. CE Angle (Degree)	Postop. CE Angle (Degree)	Preop. CCD Angle (Degree)	Postop. CCD Angle	Preop. CHTD (mm)	Postop. CHTD (mm)	Preop. ATD (mm)	Postop. ATD (mm)	Trendelenburg Sign
1	11	m	n/a	B	IV	F	R		11	19	130	117	6	8	16	16.4	no
2	5	f	yes	C	IV	C	R		19	31	142	104	5	2	16.8	14.4	yes
3	9	m	n/a	B/C	II	I	F		13	18	135	107	7	5	17	14	no
4	8	f	n/a	C	IV	C		V	14	14	145	125	7	11	17	5.8	no
5	7	m	no	C	III	F		II	9	29	143	140	6	10	19.1	11.4	yes
6	7	m	yes	B/C	IV	C	R		21	21	147	130	10	12	21	18	no
7a	6	m	n/a	C	IV	C		II	6	12	133	118	5	3	14.5	21.7	no
7b	10	m	yes	B/C	IV	F	R		23	25	131	106	3	9	24	29	no
8	7	m	yes	B/C	IV	F	R		19	32	142	116	8	4	19.8	28.7	no
9	8	m	no	B	IV	F	R		15	22	132	122	4	0	17	15	no

## Data Availability

The datasets used and/or analyzed during the current study are available from the corresponding author on reasonable request.
